# The relationship between impulsivity and decision-making styles in individuals with episodic migraine

**DOI:** 10.3389/fneur.2026.1885833

**Published:** 2026-08-12

**Authors:** Aydın Talip Yıldoğan, Dilek İşcan, Ramazan Şencan, Derviş Topuz, Fettah Eren

**Affiliations:** 1 Department of Neurology, Faculty of Medicine, Nigde Omer Halisdemir University, Nigde, Türkiye; 2 Department of Neurology, Adana Dr Turgut Noyan Training and Research Hospital, Faculty of Medicine, Baskent University, Adana, Türkiye; 3 Gaziantep City Hospital, Gaziantep, Türkiye; 4 Niğde Zübeyde Hanım Vocational School of Health Services, Department of Health Services, Niğde Ömer Halisdemir University, Niğde, Türkiye; 5 Department of Neurology, Faculty of Medicine, Selcuk University, Konya, Türkiye

**Keywords:** anxiety, decision-making styles, episodic migraine, impulsivity, maladaptive decision-making

## Abstract

**Background:**

Migraine is a complex neurological disorder that is not limited to headache attacks; it can affect cognitive, behavioral, and emotional processes. This study aims to examine whether individuals with episodic migraine and healthy controls differ in impulsivity and decision-making styles and to evaluate the relationships among these variables.

**Method:**

This cross-sectional study included patients with episodic migraine and age- and sex-matched healthy controls. All assessments were performed during the interictal period. Impulsivity was assessed using the Barratt Impulsiveness Scale (BIS-11), decision-making styles using the Melbourne Decision Making Questionnaire (MDMQ), migraine disability using the Migraine Disability Assessment Scale (MIDAS), depression using the Beck Depression Inventory (BDI), and anxiety levels using the Generalized Anxiety Disorder-7 (GAD-7). In addition to between-group comparisons, correlation, hierarchical regression, and exploratory cross-sectional indirect-association (mediation) analyses were performed. The mediation analyses were cross-sectional and exploratory.

**Results:**

A total of 200 participants (100 patients with episodic migraine and 100 healthy controls) were included. In the migraine group, levels of impulsivity (BIS-11), depression (BDI), and anxiety (GAD-7) were found to be significantly higher compared to healthy controls (*p* < 0.001 for all comparisons). The maladaptive decision-making styles of hypervigilance, procrastination, and buck-passing were significantly increased in the migraine group; the only style for which no significant between-group difference was found was vigilance (*p* = 0.098). Correlation analyses revealed a positive relationship between total impulsivity scores and procrastination (*r* = 0.478, *p* < 0.001) and buck-passing (*r* = 0.412, *p* < 0.001). Exploratory indirect-association analyses indicated that the relationships between migraine and procrastination and buck-passing were expressed as indirect statistical associations operating through impulsivity (*p* < 0.001 for both).

**Conclusion:**

Individuals with episodic migraine showed higher impulsivity, anxiety, and some maladaptive decision-making styles than healthy controls. Exploratory analyses suggest that some of the decision-making style differences observed between individuals with migraine and healthy controls may be associated with accompanying psychological characteristics such as impulsivity, rather than being attributable directly to the migraine diagnosis. However, because these findings are based on cross-sectional data, they should not be interpreted as causal mechanisms.

## Introduction

Migraine is a chronic neurological disease characterized by periodic attacks. It is also a public health problem that reduces quality of life and places a significant burden on healthcare systems. Global disease burden data reveals that migraine is one of the main causes of reduced quality of life and loss of workforce, especially in the productive age group (1). The high prevalence and chronic course of migraine create a significant clinical and socioeconomic burden (2). In recent years, it has been shown that migraine is not limited to headache attacks but is a multidimensional neurological disease that also affects cognitive and behavioral processes (3). Neuroimaging findings indicate functional changes in brain networks associated with cognitive control and behavioral regulation in migraine patients, particularly at the prefrontal cortex, anterior cingulate cortex, and limbic system levels (4). These brain networks play an important role in behavioral inhibition, risk assessment, and decision-making processes (5). Impulsivity is a multidimensional trait that refers to the tendency to act quickly and in an unplanned manner without adequately evaluating the consequences, and it is closely related to executive functions. Increased impulsivity is associated with risky decision-making and weakened behavioral control (6). It is suggested that chronic pain, stress, and neurobiological changes in migraine patients can affect behavioral control processes and alter the level of impulsivity (7). Therefore, studying the cognitive and behavioral dimensions of migraine is becoming increasingly important.

Psychiatric comorbidities such as depression and anxiety are common in migraine patients, and this can affect both disease burden and cognitive processes (8). Anxiety is a common cluster of psychological symptoms characterized by excessive and difficult-to-control worry that may be accompanied by signs of physiological arousal (9). In line with these core features, anxiety symptoms in the present study were assessed using the GAD-7, a brief and widely validated screening instrument developed to evaluate the core symptoms of generalized anxiety disorder (10). The Migraine Disability Assessment Scale (MIDAS), used to evaluate the impact of migraine on daily life, is an important clinical tool that measures the burden of the disease on work, school, and social life (11). It is thought that an increase in migraine burden can affect behavioral control and decision-making processes through chronic stress and functional impairment (3). However, studies examining the interaction between impulsivity, decision-making styles, and disease burden in individuals with migraine within a comprehensive model framework are limited in the literature. Furthermore, previous studies have suggested that medication-overuse headache may be associated in particular with increased impulsivity (12, 13), and impaired decision-making processes and functional changes in reward-processing networks have been reported in patients with chronic migraine and medication-overuse headache (14, 15). These findings suggest that chronicity and medication overuse may independently affect cognitive and behavioral processes. Therefore, to minimize these potential confounders and to evaluate whether maladaptive decision-making patterns are also present in episodic migraine, only patients diagnosed with episodic migraine were included in the study.

The main aim of this study is to examine whether individuals with episodic migraine and healthy controls differ in impulsivity and decision-making styles and to evaluate the relationships among these variables. In addition, exploratory mediation analyses were used to evaluate the observed relationships in greater detail.

## Materials and methods

### Study design

This study is a cross-sectional observational study conducted at the Neurology Outpatient Clinic of Niğde Training and Research Hospital. The study included patients diagnosed with migraine and age- and sex-matched healthy controls. Written informed consent was obtained from all participants before participation in the study. The study was conducted in accordance with the principles of the Helsinki Declaration. Approval for the study was obtained from the Ethics Committee of Niğde Ömer Halisdemir University with decision number 2025/16-49 dated 01.10.2025. Migraine diagnosis was made according to the International Classification of Headache Disorders-3 (ICHD-3) criteria (16). Assessments were not performed during an acute headache attack, but in the interictal period, at least 48 h after the last headache attack and at least 24 h before the onset of a new attack. The study was conducted between October 2025 and April 2026.

### Participants

The migraine group included individuals aged 18 and over who presented to the neurology outpatient clinic and were diagnosed with migraine according to ICHD-3 criteria. The healthy control group consisted of volunteers who attended the hospital for routine administrative health purposes, such as medical board procedures, driver’s license evaluations, and pre-employment examinations. All control participants were evaluated by a neurologist through a detailed clinical interview and neurological examination, and migraine and other primary headache disorders were excluded according to ICHD-3 criteria (16). In addition, individuals with a known history of neurological or psychiatric disease, those using psychotropic medication, or those in whom the clinical evaluation revealed a significant medical condition that could affect cognitive, behavioral, or emotional functioning were excluded. The groups were matched for age and sex. The recruitment strategy for the healthy control participants and their study-related clinical and neurological assessments were included in the study protocol reviewed and approved by the Ethics Committee.

### Exclusion criteria

Exclusion criteria for both groups were defined as chronic migraine diagnosis (headache ≥15 days per month in the last 3 months), medication overuse headache [use of triptans or combination analgesics ≥10 days per month, or simple analgesics ≥15 days per month], use of migraine prophylactic treatment in the last 3 months, pregnancy, history of psychiatric illness or use of psychotropic drugs, intellectual disability, and presence of chronic systemic disease that may affect cognitive processes (16).

### Sample size and power analysis

The required sample size was determined by *a priori* power analysis based on previous related studies evaluating impulsivity and decision-making processes in patients with episodic migraine (12, 17). An independent samples model was used to evaluate the possible difference between migraine and control groups using G*Power (v3.1.9.7) software; the effect size was accepted as Cohen’s *d* = 0.50, *α* = 0.05 and test power (1 − *β*) = 0.90 (18). As a result of the analysis, the required minimum sample size was calculated as a total of 172 participants. The total of 200 participants included in the current study (100 patients with episodic migraine and 100 healthy controls) exceeded the calculated minimum sample size.

### Data collection

Data on key demographic variables such as age, sex, and body mass index (BMI) of the participants were recorded. In the migraine group, in addition to these data, clinical parameters such as disease duration (years), migraine type (with/without aura), monthly attack frequency, and pain intensity were comprehensively evaluated. Headache intensity was scored between 0 and 10 using the Visual Analog Scale (VAS). Acute analgesic use and migraine prophylactic treatments were also assessed in migraine patients. Acute analgesic use was evaluated based on the number of days of use in the last 3 months, and individuals diagnosed with medication overuse headache according to ICHD-3 criteria were excluded from the study. Individuals who used migraine prophylactic treatment in the last 3 months were also excluded to eliminate potential pharmacological effects on behavioral and cognitive processes and to obtain a more clinically well-defined sample.

The impact of migraine on daily life was assessed using the Migraine Disability Assessment Scale (MIDAS). The MIDAS scale consists of five items that measure the number of days lost in work, home, and social life due to migraine in the last 3 months, with a total score ranging from 0 to 270 (11). The Turkish validity and reliability study of the scale was conducted by Ertaş et al. (19).

### Psychological and behavioral scales

#### Barratt Impulsiveness Scale

The level of impulsivity was assessed using the Barratt Impulsiveness Scale-11 (BIS-11). The BIS-11 is a self-report scale consisting of 30 items using a four-point Likert-type scoring system. The scale consists of three sub-dimensions: attention-related impulsivity, motor impulsivity, and lack of planning. Some items in the scale are reverse-scored, and after the reverse scoring process, the subscale and total impulsivity scores were calculated according to the original scoring guidelines of the scale (20). Both the total BIS-11 score and the subscale scores were evaluated in the analyses. The Turkish validity and reliability study of the scale was conducted by Güleç et al. (21).

#### Melbourne Decision Making Questionnaire

Decision-making characteristics were assessed using the Melbourne Decision Making Questionnaire (MDMQ). The scale was developed by Mann et al. and aims to evaluate the behavioral strategies that individuals use in their decision-making processes (22, 23). The scale consists of two parts:

- MDMQ-I: Measures decision-making self-esteem.
- MDMQ-II: Evaluates four different decision-making styles: vigilance, procrastination, buck-passing, and hypervigilance.

Subscale scores were calculated by summing the relevant items, and the evaluation was done in accordance with the original scoring guidelines of the scale (22). The Turkish adaptation of the scale was carried out by Deniz (24). In the MDMQ, higher vigilance scores indicate more effective and systematic decision-making, whereas higher hypervigilance, procrastination, and buck-passing scores indicate greater reliance on these maladaptive strategies.

#### Beck Depression Inventory

Depressive symptoms were assessed using the Beck Depression Inventory (BDI). The BDI is a 21-item self-report scale, with each item scored between 0 and 3. The total score ranges from 0 to 63, with higher scores indicating more severe depressive symptoms (25). The Turkish validity and reliability study of the scale was conducted by Hisli (26).

#### GAD-7 anxiety scale

Anxiety level was assessed using the Generalized Anxiety Disorder-7 (GAD-7). The GAD-7 is a 7-item self-report scale that measures anxiety symptoms over the past 2 weeks. Each item is scored between 0 and 3, with a total score ranging from 0 to 21 (10). The Turkish validity and reliability study of the scale was conducted by Konkan et al. (27).

#### Statistical analysis

Statistical analyses were performed using SPSS v26.0 and jamovi. Normality was assessed using the ±1.5 skewness/kurtosis criterion; all continuous variables met this criterion, and Welch’s *t*-test was used (28). Categorical variables were compared using the chi-square test. Pearson correlations were calculated. Partial correlations were computed controlling for the group variable (migraine–control); in this approach, the variance attributable to the group variable was removed from both variables, and correlation coefficients were obtained from the remaining residual variance. Factors associated with MIDAS scores were examined using hierarchical multiple linear regression, with variables entered in seven sequential blocks (Block 1: hypervigilance; Block 2: buck-passing; Block 3: GAD-7; Block 4: BIS-11 total; Block 5: sex; Block 6: age at migraine onset; Block 7: disease duration). Regression assumptions were tested for residual normality (Shapiro–Wilk), homoscedasticity (Breusch–Pagan), autocorrelation (Durbin–Watson), and multicollinearity (VIF); Benjamini–Hochberg FDR correction (*q* < 0.05) was applied to all final-model coefficients. Exploratory cross-sectional mediation analyses were performed. To evaluate whether the between-group differences were independent of depression, anxiety, and sex, a multivariate analysis of covariance (MANCOVA) was applied, controlling for BDI, GAD-7, and sex as covariates; Benjamini–Hochberg FDR correction was applied for multiple comparisons. A distinction was made between pre-planned and *post hoc* analyses. A statistical significance level of *p* < 0.05 was accepted for all analyses.

## Results

### Comparison of demographic and clinical characteristics

The study population consisted of a total of 200 participants (100 episodic migraine patients and 100 healthy controls). The migraine and control groups showed a similar distribution in terms of age, sex, and body mass index (BMI) (*p* > 0.05 for all variables). The sex distribution between the groups was equal in both groups (77% female, 23% male) and no statistically significant difference was found (*χ*^2^ = 0.00, *p* = 1.00). Demographic, clinical, and psychometric characteristics are presented in Table 1.

**Table 1:** Demographic, clinical, and psychometric characteristics of the migraine and control groups.

Variable	Control (*n* = 100)	Migraine (*n* = 100)	*p*-value	Effect size
Demographics				
Age (years)	30.24 ± 5.12	29.27 ± 5.51	0.199^t^	0.18
Sex, female, *n* (%)	77 (77)	77 (77)	1.000	–
BMI (kg/m^2^)	24.26 ± 2.47	24.78 ± 2.62	0.147^t^	0.20
Clinical characteristics (migraine group only)				
Disease duration (years)	–	7.06 ± 3.34	–	–
Monthly attack frequency	–	4.58 ± 2.15	–	–
VAS pain score	–	7.38 ± 1.07	–	–
MIDAS score	–	14.11 ± 6.77	–	–
Migraine without aura, *n* (%)	–	74 (74)	–	–
Migraine with aura, *n* (%)	–	26 (26)	–	–
Acute medication use (days/month, migraine group only)				
Analgesic use	–	2.47 ± 2.13	–	–
Triptan use	–	1.99 ± 1.61	–	–
Total acute medication use	–	4.46 ± 2.30	–	–
MIDAS disability grade (migraine group only)				
Grade I (little/no disability)	–	11 (11)	–	–
Grade II (mild)	–	22 (22)	–	–
Grade III (moderate)	–	56 (56)	–	–
Grade IV (severe)	–	11 (11)	–	–
Psychometric scales				
BDI	7.85 ± 5.06	11.90 ± 6.04	<0.001^t^	0.73
GAD-7	5.59 ± 2.94	7.63 ± 4.12	<0.001^t^	0.57
BIS-11 total	55.75 ± 8.98	62.82 ± 10.30	<0.001^m^	0.126
Attention	14.90 ± 2.77	16.74 ± 3.24	0.017ᵐ	0.086
Motor	19.39 ± 3.58	21.26 ± 4.11	0.033ᵐ	0.056
Non-planning	21.46 ± 4.27	24.82 ± 4.52	<0.001ᵐ	0.129
Decision-making styles (MDMQ)				
Vigilance	8.08 ± 1.86	7.62 ± 2.04	0.098^t^	0.23
Hypervigilance	5.44 ± 2.10	6.13 ± 2.19	0.024^t^	0.32
Procrastination	3.59 ± 2.09	4.38 ± 2.03	0.007^t^	0.38
Buck-passing	5.59 ± 2.05	6.29 ± 2.26	0.023^t^	0.32

Data are presented as mean ± standard deviation unless otherwise indicated. –, not applicable. Effect size is Cohen’s *d* for variables compared with Welch’s *t*-test and partial eta squared (*η*^2^*p*) for the variable analysed with MANCOVA. ᵗ*p* value from Welch’s *t*-test; 𝔋enjamini–Hochberg–corrected *p* value from MANCOVA with sex, BDI, and GAD-7 as covariates (not an independent-samples *t*-test); the corresponding effect size is partial eta squared (*η*^2^*p*). For the BIS-11 subscales (attention, motor, non-planning), group differences were evaluated with MANCOVA (sex as covariate); raw descriptive means are shown for reference, and the Benjamini–Hochberg–corrected *p* values and partial eta squared (*η*^2^*p*) values are reported. Categorical variables (sex, aura status) were compared with the chi-square test. BMI, body mass index; VAS, Visual Analog Scale; MIDAS, Migraine Disability Assessment Scale; BDI, Beck Depression Inventory; GAD-7, Generalized Anxiety Disorder-7; BIS-11, Barratt Impulsiveness Scale-11; MDMQ, Melbourne Decision Making Questionnaire.

In the migraine group, monthly acute medication use was as follows: mean analgesic use 2.47 ± 2.13 days/month, triptan use 1.99 ± 1.61 days/month, and total acute medication use 4.46 ± 2.30 days/month. By design, no participant met the ICHD-3 criteria for medication-overuse headache.

In the migraine group, the distribution by MIDAS disability grade was: Grade I (little/no disability) 11 patients (11%), Grade II (mild) 22 patients (22%), Grade III (moderate) 56 patients (56%), and Grade IV (severe) 11 patients (11%).

### Group-based comparison of psychometric scales and decision-making strategies

Individuals in the migraine group had significantly higher levels of depression (BDI), anxiety (GAD-7), and total impulsivity (BIS-11) than the healthy control group (*p* < 0.001 for all variables). The effect sizes for these differences were moderate (Cohen’s *d* = 0.57–0.73). The migraine group scored significantly higher than controls on all three BIS-11 subscales (attention, motor, and non-planning; see Table 1). A MANCOVA with sex as a covariate confirmed a significant multivariate group difference for the BIS-11 subscales (Wilks’ *Λ* = 0.858, *F*(3, 195) = 10.778, *p* < 0.001, *η*^2^*p* = 0.142). Multivariate normality and homogeneity of covariance were satisfied. In the Benjamini–Hochberg–corrected univariate analyses, the group difference was significant for all three subscales: attention [*F*(1, 197) = 18.562, *p* < 0.001, p_BH = 0.017], motor [*F*(1, 197) = 11.742, *p* < 0.001, p_BH = 0.033], and non-planning [*F*(1, 197) = 29.096, *p* < 0.001, p_BH < 0.001]. Effect sizes [*η*^2^*p*] and assumption-test statistics are reported in the corresponding table.

Melbourne Decision Making Questionnaire data showed that migraine patients used maladaptive decision-making strategies more frequently than the control group. The subscale scores for hypervigilance, procrastination, and buck-passing were significantly higher in the migraine group (*p* < 0.05 for all subscales). In contrast, no statistically significant difference was observed between the groups in terms of vigilance scores, an adaptive decision-making strategy (*p* = 0.098). The detailed distribution of psychometric data and group comparisons are presented in Table 1.

### Additional analyses adjusting for BDI and GAD-7

To evaluate whether the between-group differences were independent of depression and anxiety symptoms, a MANCOVA was applied with BDI and GAD-7 as continuous covariates and sex as an independent variable. When BDI and GAD-7 were statistically controlled, the group factor showed a significant multivariate effect on the set of dependent variables [*F*(5, 190) = 6.005, *p* < 0.001]. The main effect of sex [*F*(5, 190) = 1.236, *p* = 0.294] and the group × sex interaction [*F*(5, 190) = 0.557, *p* = 0.733] were not significant. Among the covariates, both BDI [*F*(5, 190) = 3.626, *p* = 0.004] and GAD-7 [*F*(5, 190) = 10.985, *p* < 0.001] showed significant multivariate effects on the set of dependent variables. Detailed multivariate statistics (Wilks’ *Λ* and η^2^p) are provided in the corresponding table.

In Benjamini–Hochberg FDR-corrected univariate analyses, the group effect remained significant for BIS-11 total impulsivity [*F*(1, 194) = 27.964, *p* < 0.001], hypervigilance [*F*(1, 194) = 6.770, *p* = 0.010], procrastination [*F*(1, 194) = 7.358, *p* = 0.007], and buck-passing [*F*(1, 194) = 5.285, *p* = 0.023]. No significant difference was found for vigilance in this adjusted analysis [*F*(1, 194) = 2.775, *p* = 0.097], consistent with the unadjusted comparison (*p* = 0.098). Examination of the univariate effects of the covariates showed that anxiety symptoms (GAD-7) were particularly influential on hypervigilance (*η*^2^*p* = 0.213) and impulsivity, whereas depressive symptoms (BDI) were mainly influential on hypervigilance (*η*^2^*p* = 0.063). The group effect remained significant with a large effect size after controlling for BDI and GAD-7. Effect sizes (*η*^2^*p*) for all univariate tests are reported in Table 2.

**Table 2:** MANCOVA of impulsivity and decision-making styles adjusting for depression, anxiety, and sex (*N* = 200).

Effect	Dependent variable	Wilks’ *Λ*	*F*	df	*p*	*η* ^2^ *p*
Panel A. BIS-11 subscales (attention, motor, non-planning)						
Group	Multivariate	0.858	10.778	3, 195	<0.001	0.142
	Attention	–	18.562	1, 197	0.017†	0.086
	Motor	–	11.742	1, 197	0.033†	0.056
	Non-planning	–	29.096	1, 197	<0.001†	0.129
Sex	Multivariate	0.992	0.556	3, 195	0.645	0.008
Panel B. BIS-11 total and decision-making styles (MDMQ)						
Group	Multivariate	0.864	6.005	5, 190	0.001	0.136
	BIS-11 total	–	27.964	1, 194	0.001†	0.126
	Vigilance	–	2.775	1, 194	0.097	0.014
	Hypervigilance	–	6.770	1, 194	0.010†	0.034
	Procrastination	–	7.358	1, 194	0.007†	0.037
	Buck-passing	–	5.285	1, 194	0.023†	0.027
Sex	Multivariate	0.969	1.236	5, 190	0.294	0.031
Group × Sex	Multivariate	0.986	0.557	5, 190	0.733	0.014

Wilks’ *Λ* is reported for multivariate effects; univariate follow-up tests are denoted –. *η*^2^*p*, partial eta squared [0.01 = small, 0.06 = medium, 0.14 = large; (36)]. For covariates, only univariate effects that retained significance after correction are listed. ^†^Significant after Benjamini–Hochberg false discovery rate correction (*k* = 5, *α* = 0.05). BDI, Beck Depression Inventory; GAD-7, Generalized Anxiety Disorder-7; BIS-11, Barratt Impulsiveness Scale-11.

### Correlation analysis

In partial correlation analyses controlling jointly for depression (BDI), anxiety (GAD-7), group, and sex, the positive relationships of impulsivity with procrastination (*r* = 0.451, *p* < 0.001), buck-passing (*r* = 0.382, *p* < 0.001), and hypervigilance (*r* = 0.216, *p* = 0.002), as well as the negative relationship with vigilance (*r* = −0.255, *p* < 0.001), remained significant. The relationship between anxiety and hypervigilance also remained significant after controlling for covariates (*r* = 0.488, *p* < 0.001). These results indicate that these relationships persisted independently of depressive and anxious symptoms and of group membership (Table 3).

**Table 3:** Partial correlation matrix of psychometric variables (*N* = 200).

Variable	1	2	3	4	5	6	7
1. BDI	–						
2. GAD-7	0.215**	–					
3. BIS-11	0.138	0.232**	–				
4. Vigilance	−0.147*	−0.065	−0.255***	–			
5. Hypervigilance	0.225**	0.488***	0.216**	0.022	–		
6. Procrastination	0.080	0.029	0.451***	−0.056	0.057	–	
7. Buck-passing	0.063	0.071	0.382***	−0.114	0.112	0.156*	–

Values are partial correlation coefficients. Columns 1–2 (BDI, GAD-7) were computed controlling for group and sex (set a; 2 covariates). Columns 3–7 (BIS-11 and MDMQ subscales) were computed controlling for BDI, GAD-7, group, and sex (set b; 4 covariates). Benjamini–Hochberg false discovery rate correction was applied within theoretically defined test families. BDI, Beck Depression Inventory; GAD-7, Generalized Anxiety Disorder-7; BIS-11, Barratt Impulsiveness Scale-11; MDMQ, Melbourne Decision Making Questionnaire. *p < 0.05; **p < 0.01; ***p < 0.001.

### Factors associated with migraine severity: hierarchical regression analysis

A seven-step hierarchical linear regression was performed to identify variables predicting MIDAS total scores (*N* = 100). Variables were entered in seven sequential blocks: hypervigilance (Block 1), buck-passing (Block 2), GAD-7 (Block 3), BIS-11 total (Block 4), sex (Block 5), age at onset (Block 6), and disease duration (Block 7). All regression assumptions were met (Shapiro–Wilk *W* = 0.989, *p* = 0.556; Breusch–Pagan *p* = 0.468; Durbin–Watson = 2.245, *p* = 0.172); VIF values remained within an acceptable range (VIF = 1.08–2.00). In Model 1, hypervigilance alone significantly explained 17.1% of the variance in MIDAS [*R*^2^ = 0.171, *F*(1, 98) = 20.17, *p* < 0.001, *β* = 0.413]. In Model 2, the addition of buck-passing increased the explained variance to 20.8%, and the model change was nominally significant [Δ*R*^2^ = 0.037, ΔF(1, 97) = 4.58, *p* = 0.035]; however, this contribution did not survive Benjamini–Hochberg FDR correction. GAD-7, BIS-11 total, sex, age at onset, and disease duration, added sequentially, provided no significant additional explanatory contribution (all *p* > 0.05). The final model (Model 7) explained 22.2% of the variance in MIDAS [*R*^2^ = 0.222, adjusted *R*^2^ = 0.163, *F*(7, 92) = 3.746, *p* = 0.001]. After Benjamini–Hochberg correction (*q* < 0.05), only hypervigilance retained statistical significance and emerged as the single independent predictor of MIDAS scores (*β* = 0.321, *p* = 0.009). Detailed results are presented in Table 4.

**Table 4:** Seven-step hierarchical regression predicting MIDAS total scores (migraine group, *n* = 100).

Step/variable added	*R* ^2^	Δ*R*^2^	ΔF (df1, df2)	*p*
Step 1 – Hypervigilance	0.171	0.171	20.17 (1, 98)	<0.001
Step 2 – Buck-passing	0.208	0.037	4.58 (1, 97)	0.035
Step 3 – GAD-7	0.210	0.002	0.26 (1, 96)	0.610
Step 4 – BIS-11 total	0.212	0.002	0.28 (1, 95)	0.600
Step 5 – Sex	0.213	0.000	0.04 (1, 94)	0.835
Step 6 – Age at onset	0.218	0.005	0.64 (1, 93)	0.426
Step 7 – Disease duration	0.222	0.004	0.43 (1, 92)	0.516

Final model (Model 7) predictor	B	SE	*β*	95% CI [LL, UL]	*p*
Hypervigilance	0.991	0.370	0.321	[0.256, 1.727]	0.009
Buck-passing	0.567	0.323	0.189	[−0.075, 1.208]	0.083
GAD-7	0.092	0.193	0.056	[−0.292, 0.476]	0.635
BIS-11 total	0.028	0.073	0.043	[−0.117, 0.173]	0.702
Sex (F – M)	−0.410	1.530	−0.061	[−3.448, 2.628]	0.789
Age at onset	0.041	0.308	0.017	[−0.572, 0.653]	0.895
Disease duration	0.178	0.273	0.085	[−0.365, 0.721]	0.516

*B*, unstandardized coefficient; SE, standard error; *β*, standardized coefficient; CI, confidence interval; LL, lower limit; UL, upper limit. Regression assumptions were satisfied (Shapiro–Wilk *W* = 0.989, *p* = 0.556; Breusch–Pagan *p* = 0.468; Durbin–Watson = 2.245; VIF = 1.08–2.00). Benjamini–Hochberg false discovery rate correction was applied to the final-model coefficients; only hypervigilance retained significance. MIDAS, Migraine Disability Assessment Scale; GAD-7, Generalized Anxiety Disorder-7; BIS-11, Barratt Impulsiveness Scale-11. Model summary: *R*^2^ = 0.222, adjusted *R*^2^ = 0.163, *F*(7, 92) = 3.746, *p* = 0.001.

### Additional analyses

The effect of sex was evaluated in the MANCOVA model as both a main effect and a group × sex interaction; no significant interaction was found at the multivariate level (Wilks’ *Λ* = 0.986, *p* = 0.733). At the level of individual outcome variables, none of the group × sex interactions were significant (all *p* > 0.22).

To evaluate the relationship of clinical characteristics with impulsivity in the migraine group, a multiple linear regression (enter method) was performed to identify clinical predictors of BIS-11 total impulsivity (*N* = 100). Presence of aura (yes/no), monthly attack frequency, pain severity (VAS), and disease duration were entered simultaneously. Residuals were normally distributed (Shapiro–Wilk *W* = 0.989, *p* = 0.562), variance was homogeneous (Breusch–Pagan *p* = 0.549), and no autocorrelation was present (Durbin–Watson = 2.176, *p* = 0.374); VIF values remained within an acceptable range (VIF = 1.01–1.27). With a maximum Cook’s distance of 0.071, no influential outliers were detected.

The model was statistically significant [*F*(4, 95) = 3.254, *p* = 0.015] and explained 12.1% of the variance in BIS-11 total scores (adjusted *R*^2^ = 0.083). Monthly attack frequency was a significant predictor of BIS-11 total (*β* = 0.225, *p* = 0.039), whereas presence of aura (*β* = 0.094, *p* = 0.670), pain severity (*β* = 0.172, *p* = 0.117), and disease duration (*β* = 0.059, *p* = 0.551) were not significant predictors. Detailed results are presented in Table 5.

**Table 5:** Multiple linear regression of clinical predictors of BIS-11 total impulsivity (migraine group, *n* = 100).

Predictor	*B*	SE	*β*	95% CI [LL, UL]	*p*
Constant	44.123	7.086	–	–	<0.001
Aura (present – absent)	0.964	2.256	0.094	[−0.341, 0.528]	0.670
Monthly attack frequency	1.080	0.516	0.225	[0.012, 0.439]	0.039
Pain severity (VAS)	1.652	1.045	0.172	[−0.044, 0.387]	0.117
Disease duration	0.187	0.312	0.059	[−0.135, 0.253]	0.551

*B*, unstandardized coefficient; SE, standard error; *β*, standardized coefficient; CI, confidence interval (for *β*); LL, lower limit; UL, upper limit. Model: *R*^2^ = 0.121, Adjusted *R*^2^ = 0.083, *F*(4, 95) = 3.254, *p* = 0.015. Assumptions satisfied (Shapiro–Wilk *W* = 0.989, *p* = 0.562; Breusch–Pagan *p* = 0.549; Durbin–Watson = 2.176; VIF = 1.01–1.27; maximum Cook’s distance = 0.071). BIS-11, Barratt Impulsiveness Scale-11; VAS, Visual Analog Scale.

### Mediation analyses

The relationships among migraine diagnosis, impulsivity, and maladaptive decision-making styles were evaluated using exploratory indirect-association analyses. In the migraine → impulsivity → procrastination model, the indirect effect was statistically significant (standardized *β* = 0.162, 95% CI [0.090–0.245], *p* < 0.001). Similarly, in the migraine → impulsivity → buck-passing model, the indirect effect was significant (standardized *β* = 0.127, 95% CI [0.060–0.215], *p* < 0.001). In both models, while the total effect was significant (*p* = 0.006 and *p* = 0.020, respectively), the direct effect between migraine and the relevant decision-making style lost statistical significance once impulsivity was included as an intervening variable (*p* = 0.676 and *p* = 0.861, respectively).

**Figure 1:**
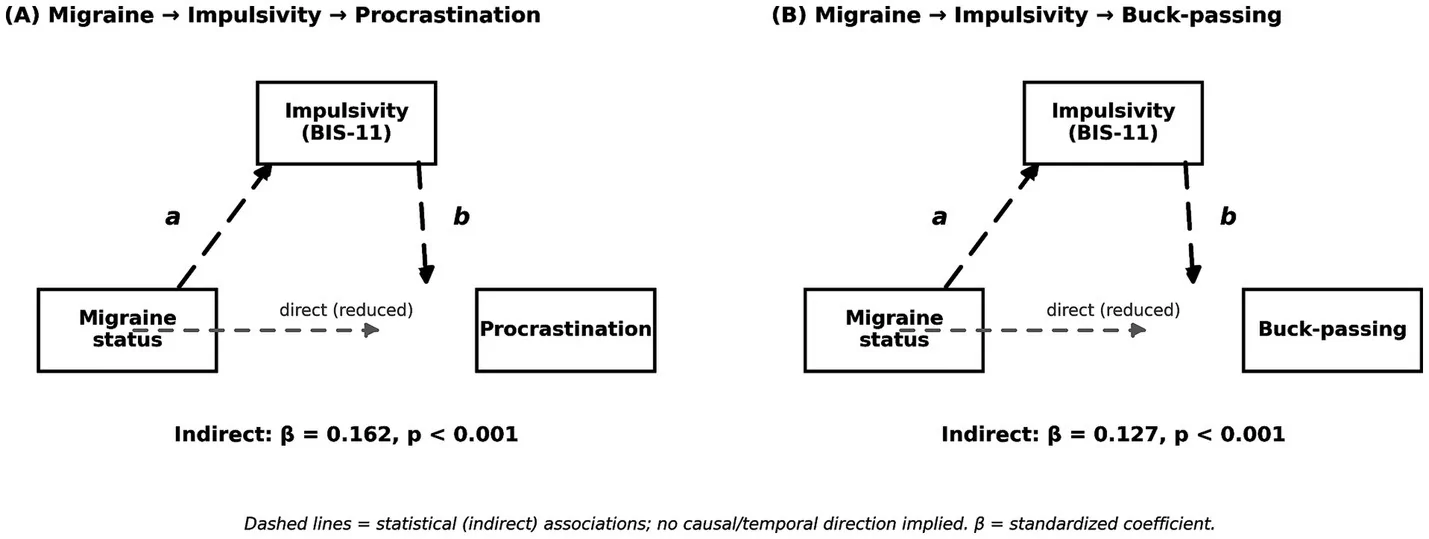
Exploratory cross-sectional indirect-association models linking migraine status, impulsivity, and two maladaptive decision-making styles. These analyses are exploratory and represent one component of the broader set of descriptive and inferential analyses reported in the study (group comparisons, correlation, partial correlation, MANCOVA, and regression; see Tables 1–5). **(A, B)** The association between migraine status and procrastination and **(B)** the association between migraine status and buck-passing, each examined in relation to impulsivity (BIS-11) as an intervening variable. In each model, path a denotes the migraine–impulsivity association, path *b* the impulsivity–outcome association, and the grey arrow the direct association between migraine status and the outcome after the intervening variable is included in the model. The standardized indirect coefficients were *β* = 0.162 (95% CI [0.090, 0.245], *p* < 0.001) for procrastination and *β* = 0.127 (95% CI [0.060, 0.215], *p* < 0.001) for buck-passing; in both models the direct association became non-significant once impulsivity was included. Dashed lines denote statistical (indirect) associations and do not imply any causal or temporal direction. Confidence intervals were obtained by percentile bootstrapping and indirect effects remained significant after Benjamini–Hochberg false discovery rate correction. *β* = standardized coefficient; BIS-11 = Barratt Impulsiveness Scale-11.

The standardized coefficients and 95% confidence intervals for these exploratory indirect-association analyses are presented in Table 6, and the corresponding path diagram is shown in Figure 1.

**Table 6:** Summary of exploratory cross-sectional indirect-effect (mediation) analyses (*N* = 200).

Model	Effect	*β*	*p*	95% CI [LL, UL]	Path
Model 1: Migraine → BIS-11 → Procrastination	Indirect	0.162	<0.001	[0.090, 0.245]	Migraine⇒BIS-11 ⇒ Procrastination
	Direct	0.028	0.676	[−0.115, 0.166]	Migraine⇒Procrastination
	Total	0.189	0.006	[0.035, 0.333]	Migraine⇒Procrastination
Model 2: Migraine → BIS-11 → Buck-passing	Indirect	0.127	< 0.001	[0.060, 0.215]	Migraine⇒BIS-11 ⇒ Buck-passing
	Direct	0.012	0.861	[−0.140, 0.156]	Migraine⇒Buck-passing
	Total	0.161	0.020	[0.018, 0.301]	Migraine⇒Buck-passing

*β*, standardized effect; CI, confidence interval; LL, lower limit; UL, upper limit. Both the standardized coefficients and the 95% confidence intervals are standardized; confidence intervals were obtained from percentile bootstrapping and converted to the standardized metric. Group was coded migraine = 1, control = 0. Sex was included as a moderator of the BIS-11 → outcome path and was non-significant in both models. Indirect effects remained significant after Benjamini–Hochberg false discovery rate correction. These analyses are cross-sectional and exploratory and do not establish temporal or causal precedence. BIS-11, Barratt Impulsiveness Scale-11.

## Discussion

This study demonstrated that episodic migraine patients had significantly higher levels of depression, anxiety, and impulsivity compared to healthy controls. Furthermore, maladaptive decision-making styles such as hypervigilance, procrastination, and buck-passing were significantly increased in the migraine group; however, no significant difference was found between the groups in terms of vigilance, an adaptive decision-making style. Moreover, after controlling for BDI, GAD-7, and sex, the observed group differences in impulsivity, procrastination, hypervigilance, and buck-passing remained significant. These findings suggest that migraine constitutes a multidimensional clinical and behavioral picture involving cognitive control, behavioral regulation, and emotional processes, not limited solely to pain attacks.

When studies evaluating impulsivity in migraine patients are examined, the literature shows a heterogeneous picture. In the study by Husni et al., which included only female migraine patients, impulsivity levels were shown to be significantly higher; depression levels were also found to be significantly increased compared to the control group (7). The fact that similar findings were obtained in a mixed-sex sample including both female and male participants in the present study, with no significant group × sex interaction (*p* = 0.733), suggests that this pattern may not be unique to women. However, in the aforementioned study, impulsivity was reported only as a group difference in absolute scores; a relational model with decision-making styles and disease burden was not established. The present study, on the other hand, offers a more holistic perspective to the literature by evaluating this relationship together with decision-making styles and migraine disability.

In contrast, in the study by Santos et al., no significant difference was found in terms of impulsivity between the migraine, medication overuse headache, and control groups (12). However, impulsivity scores were numerically higher in the migraine group than in the control group. Nevertheless, differences in the samples and methods of the two studies call for caution in directly comparing their findings. In addition, no exclusion criteria were specified for treatments affecting the serotonergic system, and no distinction was made between chronic and episodic migraine. Notably, the fact that the highest impulsivity score was found in the migraine group, not the medication overuse group, in the aforementioned study suggests that impulsivity may not be related solely to medication overuse or chronicity (12).

In the study conducted by Şimşekoğlu et al. on patients with chronic migraine and medication overuse headache, impulsivity levels were shown to be significantly increased (13). However, the fact that only the chronic patient group was evaluated limits the direct generalization of the results to episodic migraine patients. The high levels of impulsivity found in the episodic migraine group in the current study suggest that this characteristic may not simply represent a consequence of chronicity; it may be one of the behavioral characteristics that can be observed even in the period before the disease becomes chronic.

In the systematic review by Gonzaga et al., it was stated that the current evidence is not yet sufficient to support a consistent relationship between migraine and impulsivity; it was emphasized that this heterogeneity may stem from differences in samples, the scales used, and inadequacies in clinical subgroup distinctions (29).

In this respect, one of the important methodological strengths of the current study is that only episodic migraine patients were included, individuals with chronic migraine and medication overuse headache were excluded, and all evaluations were performed in the interictal period. Thus, the potential confounding role of acute pain, intensive analgesic use, and chronicity-related behavioral effects has been significantly reduced. Furthermore, the fact that impulsivity and decision-making styles have been examined together with migraine disability within a relational model is considered to be a unique contribution of this study, given the limited number of methodologically comparable studies in the literature.

Most studies examining the relationship between migraine and decision-making processes rely on experimental cognitive tasks. In a study by Wang et al. on patients with episodic migraine without aura, an increased tendency toward impulsive decision-making was shown using a delayed reward discounting task; furthermore, functional connectivity changes were detected in brain regions associated with reward processing and decision-making (17). However, the absence of clinical burden measures such as MIDAS in the study and the failure to clearly exclude medication overuse make it difficult to differentiate whether the observed increase in impulsivity is due to migraine itself or to disability and medication use patterns. In a study by Biagianti et al. on patients with chronic migraine and medication overuse headache, significant decision-making impairment was demonstrated using the Iowa Gambling Task; Niddam et al. also detected functional changes in brain networks associated with reward processing in a similar clinical group (14, 15). However, the focus of both studies on chronic and more severe clinical groups limits the direct application of these findings to patients with episodic migraine. In the present study, the finding that some maladaptive decision-making styles were also higher in the migraine group suggests that differences in decision-making processes may not be confined to individuals with chronic migraine or medication-overuse headache.

In the present study, decision-making processes were assessed through the Melbourne Decision Making Questionnaire (MDMQ), unlike experimental cognitive tasks, and the decision-making patterns exhibited by individuals in daily life were measured (22). The fact that all three maladaptive styles were significantly increased in the migraine group and no significant difference was observed in terms of vigilance suggests that migraine may be associated with a selective increase in maladaptive patterns rather than a global impairment of decision-making capacity. In particular, the association of some maladaptive styles with migraine disability suggests that these styles may have clinical implications for daily living functioning and disease burden. This suggests that the assessment of behavioral and cognitive processes in migraine patients may be important not only theoretically but also from a clinical management perspective.

Although significant differences in decision-making styles were found between the groups, it is not clear whether these differences arise directly from the migraine diagnosis or from psychological characteristics more frequently observed in the migraine group. Therefore, in the exploratory mediation analyses performed, the relationships between migraine and maladaptive decision-making patterns were evaluated in the context of impulsivity. In these analyses, procrastination and buck-passing behaviors showed significant indirect statistical associations through impulsivity (*p* < 0.001 for both). The fact that the direct relationship between migraine and these decision-making styles largely lost its statistical significance once impulsivity was included in the model suggests that these patterns may not be explained by the migraine diagnosis alone. These findings point to the importance of jointly considering impulsivity, depression, anxiety, and other psychological characteristics when evaluating decision-making differences in individuals with migraine.

In addition to the exploratory mediation analyses, a regression analysis revealed another finding of potential clinical relevance: hypervigilance showed an association with migraine-related disability that was independent of the other variables. Although the direction of this association cannot be determined because of the cross-sectional design, this finding suggests that hypervigilance may show a clinically noteworthy association with disease burden in patients with migraine.

Group differences in procrastination, hypervigilance, and buck-passing remained significant after controlling for BDI and GAD-7. This indicates that these differences cannot be explained by depression and anxiety alone. At the same time, the cognitive and emotional mechanisms underlying decision-making processes may not be limited to the variables assessed in this study. Indeed, differences in alexithymia, social cognition, and theory of mind have also been reported in migraine patients (30–32). In non-migraine populations, alexithymia has been shown to be associated with decision-making processes (33), and impairments in social cognition and theory of mind have similarly been associated with impulsivity and decision-making behavior (34). These findings raise the possibility that these constructs may contribute to the impulsivity and decision-making patterns observed in migraine. Future studies that jointly assess these constructs may contribute to a more comprehensive understanding of the cognitive and psychological mechanisms underlying decision-making in migraine.

In the study by Genizi et al. on pediatric and adolescent migraine patients, executive functions and psychological processes were found to differ from those of healthy controls, and these processes were associated with social functioning and quality of life (35). Although the study population and assessed domains differ from ours, it is conceivable that the association of migraine with cognitive, psychological, and social patterns may not be specific to a particular age period and may emerge across different developmental stages (35).

The findings of this study should be interpreted in light of several limitations. The single-center design limits generalizability. Only self-report questionnaires were used; the absence of objective neuropsychological tests or laboratory-based behavioral tasks carries a potential risk of response bias, and the possibility that the comprehensive multi-item battery may have caused response fatigue cannot be excluded. Due to its cross-sectional design, the causal direction cannot be determined; alternative directional explanations—such as stress associated with procrastination increasing impulsivity or triggering migraine attacks—are equally plausible with these data. While the inclusion of only episodic migraine patients provides a more clinically well-defined sample by reducing variability related to chronicity and medication overuse, it limits the generalization of the findings to individuals with chronic migraine and medication overuse headache. In addition, the lack of comprehensive assessment of all sociodemographic variables, such as income, education, and occupational status, is another limitation. Future longitudinal and neuroimaging-based studies are expected to more clearly reveal the nature of these relationships.

## Conclusion

Compared with healthy controls, individuals with episodic migraine showed higher impulsivity and anxiety levels and greater reliance on certain maladaptive decision-making tendencies (procrastination, buck-passing, and hypervigilance). These differences were largely preserved after controlling for comorbid depressive and anxious symptoms and sex. Exploratory analyses suggest that some of the observed decision-making style differences may be associated with accompanying psychological characteristics such as impulsivity, rather than being attributable directly to the migraine diagnosis. However, because these findings are based on cross-sectional data, they should not be interpreted as causal mechanisms; the direction and stability of these relationships should be examined in further research.

## Limitations

The findings of this study should be interpreted in light of two main limitations. First, the generalizability of the results is constrained by the study’s single-center design, which was conducted in a specific region and may not fully reflect the diverse characteristics of the broader episodic migraine population. Second, the evaluation of behavioral and cognitive dimensions relied exclusively on self-report questionnaires; the absence of objective neuropsychological testing or laboratory-based behavioral tasks introduces a potential risk of response bias. Future multi-center longitudinal studies incorporating objective neurocognitive paradigms alongside clinical assessments are required to validate and expand upon these findings.

## Data Availability

The raw data supporting the conclusions of this article will be made available by the authors, without undue reservation.
